# The IL-6/JAK/STAT3 Axis in Cholangiocarcinoma and Primary Sclerosing Cholangitis: Unlocking Therapeutic Strategies Through Patient-Derived Organoids

**DOI:** 10.3390/biomedicines13051083

**Published:** 2025-04-29

**Authors:** Corinna Boden, Laura K. Esser, Leona Dold, Bettina Langhans, Taotao Zhou, Dominik J. Kaczmarek, Maria A. Gonzalez-Carmona, Tobias J. Weismüller, Glen Kristiansen, Jörg C. Kalff, Michael Hölzel, Hanno Matthaei, Marieta I. Toma, Vittorio Branchi

**Affiliations:** 1 Department of General, Abdominal, Thoracic and Vascular Surgery, University Hospital Bonn, 53127 Bonn, Germany; corinna.boden@ukbonn.de (C.B.); joerg.kalff@ukbonn.de (J.C.K.); hanno.matthaei@ukbonn.de (H.M.); 2 Institute of Pathology, University Hospital Bonn, 53127 Bonn, Germanyglen.kristiansen@ukbonn.de (G.K.);; 3 Department of Internal Medicine I, University Hospital Bonn, 53127 Bonn, Germany; leona.dold@ukbonn.de (L.D.); bettina.langhans@ukbonn.de (B.L.); taotao.zhou@ukbonn.de (T.Z.); dominik.kaczmarek@ukbonn.de (D.J.K.); maria.gonzalez-carmona@ukbonn.de (M.A.G.-C.); 4 Vivantes Humboldt-Klinikum, 13509 Berlin, Germany; tobias.weismueller@vivantes.de; 5 Institute of Experimental Oncology, University Hospital Bonn, 53127 Bonn, Germany; michael.hoelzel@ukbonn.de

**Keywords:** biliary tract cancer, cholangiocarcinoma, primary sclerosing cholangitis, STAT3, baricitinib

## Abstract

**Background/Objectives:** Primary sclerosing cholangitis (PSC) is a rare, incurable liver disease characterized by chronic biliary inflammation and fibrosis. PSC is a significant risk factor for biliary tract cancer (BTC). This study aims to evaluate STAT3 expression in BTC and its prognostic significance as well as explore the potential of organoids derived from PSC and liver tumor patients as an in vitro model for testing novel therapeutic strategies in both PSC and BTC. **Methods:** Fresh tissue samples obtained from 10 PSC patients through targeted endoscopic retrograde cholangiography (ERC) and biopsy samples from liver tumor patients were used to establish organoid cultures. Organoids were treated with different agents and the therapeutic effect was measured by CellTiterGlo. Treatment with the JAK inhibitor baricitinib was followed by the measurement of cytokine concentrations in the supernatant. Archived formalin-fixed paraffin-embedded (FFPE) samples from 55 surgically resected BTC tumors were analyzed for STAT3 expression using immunohistochemistry. **Results:** We successfully established organoid cultures from all ERC samples. STAT3 protein expression was detected in 56% of tumor samples and 69% of the immune microenvironment. STAT3 positivity in the immune cell compartment was associated with longer disease-free survival, although the multivariate analysis could not confirm its value as an independent prognostic factor. Chemotherapy testing on liver tumor organoids showed various degrees of decreases in viability after treatment with gemcitabine, cisplatin, and cabozantinib. Baricitinib treatment significantly reduced IL-6 and MCP-1 secretion in cholangiocarcinoma **Conclusions:** The patient-derived organoid model of PSC and liver tumors is a valuable tool for testing novel and established therapeutic strategies, including JAK inhibitors and chemotherapy regimens. STAT3 expression in the immune microenvironment of BTC may serve as a prognostic marker. Further studies are needed to explore the integration of co-cultured organoid systems with stromal and immune components to improve physiological relevance.

## 1. Introduction

Primary sclerosing cholangitis (PSC) is a rare, incurable, cholestatic liver disease characterized by chronic, progressive biliary inflammation and subsequent fibrosis [1]. The etiopathogenesis of PSC remains unclear and effective therapy is lacking [2]. The incidence of PSC varies in different parts of the world and is estimated to range from 0 to 1.58 per 100,000, whereas the median prevalence ranges between 0 and 24.99 per 100,000 [3]. Men have almost twice the risk of developing PSC as women [4]. PSC is found to co-exist with inflammatory bowel disease (IBD) in 60–80% of cases, with a higher prevalence of IBD in male PSC patients compared to females [5]. On the other hand, PSC occurs in approximately 8% of individuals with IBD, and the most common IBD diagnosed in PSC patients is ulcerative colitis [6]. PSC is considered a premalignant condition and represents a risk factor for various abdominal malignancies, which account for 40–50% of all deaths in patients with PSC [7,8,9,10]. Biliary tract cancer (BTC), in particular cholangiocarcinoma (CCA), is the most common malignancy and the leading cause of death in patients with PSC [11]. Early detection of BTC in PSC remains a major clinical challenge, and 1-year mortality reaches 80% in patients diagnosed with PSC-associated BTC [11]. Endoscopic retrograde cholangiography (ERC) is a crucial tool for the diagnostic evaluation and treatment of high-grade strictures in PSC. However, its use should be reserved for cases with unclear diagnoses or when tissue sampling or endoscopic interventions are necessary [12]. The limited understanding of PSC pathogenesis and the scarcity of effective therapeutic options highlight the critical need for translational research in this area.

Biliary tract cancer (BTC) refers to a heterogeneous group of aggressive, malignant tumors that arise along the bile ducts (cholangiocarcinoma—CCA) and in the gallbladder or cystic duct (gallbladder carcinoma—GBC) [13]. Most individuals with BTC receive a diagnosis at an advanced, unresectable stage, primarily because symptoms are typically absent during the initial phases of the disease [14]. BTCs are further classified as intrahepatic CCA (iCCA), which arises from bile ducts proximal to the second-order bile ducts; perihilar CCA (phCCA), which arises at the junction of the right and left hepatic ducts or within the right or left hepatic duct; distal CCA (dCCA), which arises distal to the cystic duct insertion [15]. In most countries, CCA remains a relatively rare malignancy; however, its incidence and mortality rates have steadily risen over the past few decades [16]. Incidence and mortality rate, as well as risk factors for CCA, vary substantially based on geographic regions [17]. In region where liver fluke is endemic, such as Thailand, CCA age-standardized incidence can reach 115 per 100,000 in men and 49 per 100,000 in women [18]. In regions where liver fluke is not considered endemic, the CCA incidence ranges between 0.3 and 6 per 100,000 [19]. Among the various risk factors for CCA—such as cirrhosis, cholelithiasis/choledocholithiasis, hepatitis, liver flukes, bile duct cysts, and Caroli’s disease—primary sclerosing cholangitis (PSC) stands out as the most significant risk factor in western countries [17,20]. GBC is a rare tumor with a global age-standardized incidence rate of 0.9 for males and 1.4 for females [21]. Risk factors for GBC include cholelithiasis, obesity, and bile duct infections [22].

Signal transducer and activator of transcription 3 (STAT3) is a transcription factor that belongs to the STAT family and plays a role in normal cellular processes, such as cell survival and proliferation [23]. In cancer, STAT3 overexpression has been observed in many solid tumors, as well as in hematologic malignancies, correlating with unfavorable clinical stages, and the worst overall and disease-free survival [24,25,26,27]. Hyperactivation of the STAT3 pathway contributes to key cancer hallmarks, including epithelial-to-mesenchymal transition (EMT), angiogenesis, and metastasis [28]. STAT3 signaling is initiated when cytokines or growth factors (e.g., IL-6, IL-10, EGF, PDGF, TNF) bind to their receptors, activating receptor-associated Janus kinases (JAKs). Activated JAKs phosphorylate each other and the receptor’s tyrosine residues, creating docking sites for STAT3. STAT3 is recruited and phosphorylated by JAKs, enabling its dimerization. The STAT3 dimer translocates to the nucleus, binds to specific DNA sequences, and activates transcription of a broad number of genes, including cancer promoting genes [29,30]. STAT3 has been identified as a key gene involved in all three main human cholangiopathies, including PSC [31]. In BTC, STAT3 overexpression has been associated with worse pathological characteristics, as well as negative surgical outcomes, and some data identified STAT3 as a driver of cancer proliferation and metastasis [32,33].

This study aimed to evaluate STAT3 expression in BTC and assess its prognostic significance in a cohort of BTC patients. In addition, it aimed to establish a biobank of organoids derived from both PSC and liver tumor patients to explore their potential as an in vitro model for testing novel therapeutic strategies for PSC and BTC. In this study, we aimed to use cholangioscopy-guided biopsies of the main strictures of PSC patients to provide a highly specific and minimally invasive system for organoid generation. Biopsies taken directly from the site of the main stricture may provide a targeted and clinically relevant approach for PSC organoid generation.

## 2. Materials and Methods

### 2.1. FFPE Samples from Surgically Resected Tumors

We included archived samples from patients who underwent surgical resection for BTC at the Department of Surgery. Patients’ demographic data, including gender, age, and tumor and treatment-related data, were collected. Survival data and pathological characteristics were retrieved from patients’ records. All tumors were thoroughly restaged according to the TNM classification, 8th Edition.

### 2.2. Immunohistochemistry

Antibodies to STAT3 and CK7 were used. Briefly, 3 µm sections from FFPE blocks were mounted on Tomo^®^ Adhesion microscope slides, (Matsunami Glass Ind. LTD, Osaka, Japan). The sections were deparaffinized with xylene (2 × 15 min, VWR International, Fontenay-sous-Bois, France) and rehydrated using decreasing concentrations of graded ethanol (Berkel AHK, Ludwigshafen, Germany) to water (B. Braun, Melsungen, Germany). Antigen retrieval was achieved by boiling the slides in citrate buffer (Zytomed Systems GmbH, Berlin, Germany) at pH 6.0 for 20 min. The tissue samples were then stained overnight at 4 °C. Immunohistochemistry was performed using the semi-automated platform Autostainer 480 S (Medac, Wedel, Germany). All supplementary reagents were provided by Medac.

### 2.3. Analysis of Immunoreactivity

A Tissue microarray (TMA) was constructed according to standardized protocols from FFPE blocks. First, standard hematoxylin–eosin (H&E) staining of 3 µmsection was obtained to select tumor regions. Four to six 1 mm cores per sample were transferred to the TMA. STAT3 protein expression, both in immune cells and in tumor cells, was evaluated. An expert pathologist double-checked the assessment of immune reactivity. In the immune cells compartment, the presence of a membranous or a cytoplasmic staining was considered a positive immunoreactivity. A semi-quantitative scoring system was applied, where samples were classified as STAT3-positive if more than 10% of immune cells showed positivity for STAT3. In tumor cells, STAT3 expression was evaluated as the percentage of positive cells. Mean values were calculated for each tumor. Tumor samples with less than 10% positive tumor cells were considered STAT3-negative.

### 2.4. Fresh Tissue Samples from PSC Patients and from Patients with Liver Malignancies

Biopsies from patients with PSC who underwent an elective endoscopic retrograde cholagiography (ERC) were obtained. The indication for ERC was made according to clinical evaluation and independently from this study. The ERC was performed according to clinical standards by highly experienced endoscopists. Cholangioscopies were performed with single-use, single-operator-controlled devices (SpyScopeTM DS, Boston Scientific, Marlborough, IL, USA). Biopsies for routine pathological examinations were made according to clinical evaluations. Two extra samples were obtained at the site of pathological strictures with a single-use, biopsy forceps with a 0.8 mm-wide oval spoon-shaped mouth with tooth (Micro Byte, MTW-Endoskopie, Wesel, Germany) or with a standard 2.3 mm biopsy forceps with oval cups with spikes (Endo-Flex GmbH, Voerde, Germany). The samples were directly placed in a 15 mL falcon tube filled with ice-cold Dulbecco’s PBS after collection. Within 30 min after collection, the samples were processed to obtain the extrahepatic cholangiocyte organoids (ECOs). Biopsy from surgery specimens were obtained from patients with liver tumors who underwent liver resection. The indication for surgical resection was made independently from this study through an interdisciplinary tumor board. Within 30 min of collection, the samples were then processed to obtain patient-derived organoids. Every patient included in this study signed the informed consent prior to the investigation/operation according to our institutional recommendations and in compliance with the Declaration of Helsinki.

### 2.5. Culture of Extrahepatic Cholangiocyte Organoid from PSC Patients and Tumor Organoids Derived from Primary or Metastatic Tumors of the Liver

Patient-derived organoids were cultured using an adaptation of previously described methods [34]. Fresh tissue biopsies and biopsy-like samples from surgical specimens were washed in wash media (Supplementary Table S1), minced, and incubated at 37 °C with a digestion solution (Supplementary Table S2). After 30 min of incubation, the suspension was washed three times with the wash media, filtered through a 100 μm nylon cell strainer, and spun for 5 min at 300 G. The pellet was washed in cold Dulbecco’s PBS, then mixed with Geltrex matrix (Gibco, Thermofisher, Wahltam, MA, USA) and plated as domes in a pre-warmed 6-multi-well plate. After the 15 min incubation (37 °C, 5% CO_2_), 1.5 mL of organoid isolation media (Supplementary Table S4) was added into each well. Organoid cultures were incubated at 37 °C with 5% CO_2_, and media were replaced twice a week. After one to two weeks, depending on the growth pattern, organoid isolation media were replaced with organoid expansion media (Supplementary Table S5), or organoid tumoroid media for HCC organoids (Supplementary Table S6). Once they reached a considerable density or dimension, organoids were passaged by mechanical dissociation into small fragments, transferred to fresh Geltrex matrix, and replated as domes in pre-warmed 6-multi-well plates. A mycoplasma PCR test was performed every 4 weeks. Control organoids were generated from bile duct epithelial tissue obtained from a non-diseased gallbladder, which served as external controls in the cytokine secretion experiments.

### 2.6. Organoid Treatment with a JAK Inhibitor and Measurement of Cytokines in the Organoid Supernatant

Cholangiocyte organoids were treated with a reversible Janus kinase JAK1 and JAK2 inhibitor (baricitinib). First, organoids were cultivated at least 1 week before starting the treatment. Organoids were then passaged and seeded in a 96-well plate (Sarstedt AG & Co. KG, Nümbrecht, Germany). After 72 h of incubation, organoids were treated with baricitinib at different concentrations (1 nM, 10 nM, and 100 nM). The supernatant was then collected and centrifuged to remove debris. Cytokine concentrations in supernatants were then measured through flow cytometry using the LEGENDplex HU Essential Immune Response Panel (Biolegend, San Diego, CA, USA). Samples were analyzed on a FACSCanto II (BD Biosciences, Heidelberg, Germany). Data evaluation was performed using LEGENDplex data analysis software (Biolegend, San Diego, CA, USA, https://www.biolegend.com/fr-fr/immunoassays/legendplex, accessed on 15 January 2025). In total, 13 different cytokines were analyzed: IL-4, IL-2, CCL10 (IP-10), IL-1β, TNF-α, TGF-β1, CXCL8 (IL-8), CCL2 (MCP-1), IL-17A, IL-6, IL-10, IFN-γ, and IL-12.

### 2.7. DNA Sequencing

Whole genomic DNA was extracted from cultured organoids using the innuCONVERT bisulfite all-in-one kit (Analytik Jena, Jena, Germany). Multiplex amplicon preparation and subsequent library construction were performed using the TruSight Tumor 15 Panel (Illumina, San Diego, CA, USA). The amplification products were deep-sequenced on the MiSeq (Illumina, San Diego, CA, USA) with a sequence coverage of >500 reads. VariantStudio 3.0 (Illumina, San Diego, CA, USA) was used for data analysis and identification of genomic variants. All relevant somatic mutations with an allele frequency >5% were reported. The current versions of the following online databases were used for classification and reporting of somatic variants: dbSNP, ClinVar, OncoKB, and ExAc.

### 2.8. Chemotherapy Testing

Patient-derived organoids from malignant primary liver tumors (N = 3) and liver metastasis (N = 2) were cultivated for 1 week before starting the treatment. The organoids were then passaged and seeded in a 96-well plate (Sarstedt, Germany). After 72h, organoids were treated with gemcitabine, cisplatin, or cabozantinib, either alone or in combinations, at different concentrations. Gemcitabine was used at the following concentrations: 5 µM, 10 µM, 20 µM, and 40 µM. Cisplatin working concentrations were 2.5 µM, 5 µM, 10 µM, and 20 µM. Cabozantinib was used at 4.5 µM, 9 µM, 18 µM, and 36 µM. A combination of gemcitabine and cisplatin (5/2.5 µM, 10/5 µM, 20/10 µM, 40/20 µM) was also used. After 72 h, 100-μL CellTiter-Glo^®^-Luminescent reagent (Promega, Madison, WI, US) per well was added and incubated for 20 min. The medium was then transferred to a nontransparent 96-well plate (Greiner Bio-One GmbH, Frickenhausen, Austria) and luminescence was assessed on a Centro LB 960 microplate luminometer (Berthold Technologies GmbH & Co. KG, Bad Wildbad, Germany). Every experiment was carried out as a triplicate.

### 2.9. Statistical Analysis

Statistical analysis was performed in the R environment (RStudio Version 12, packages: ggplot, survival, survminer, gt, and gtsummary) [35,36,37,38,39,40,41] and Graphpad Prism (Version 10, GraphPad Software, Boston, MA, USA). The Wilcoxon rank sum test, Pearson’s chi-squared test, and Fisher’s exact test were used as appropriate. A log-rank test was used to compare Kaplan–Meier curves. A *p* value < 0.05 was considered significant.

## 3. Results

### 3.1. Establishment of a Patient-Derived Organoid Biobank for PSC

Biopsies from ERC were obtained from ten consecutive patients, five females (50%) and five males (50%). The median age at PSC diagnosis was 37 (interquartile range 23–47). The median age of the patients at the time of the sampling was 45 years (interquartile range 39–49). In two cases, the samples were obtained at the patient’s first endoscopic retrograde cholangiography (ERC). Patient characteristics are summarized in Table 1.

**Figure 1:**
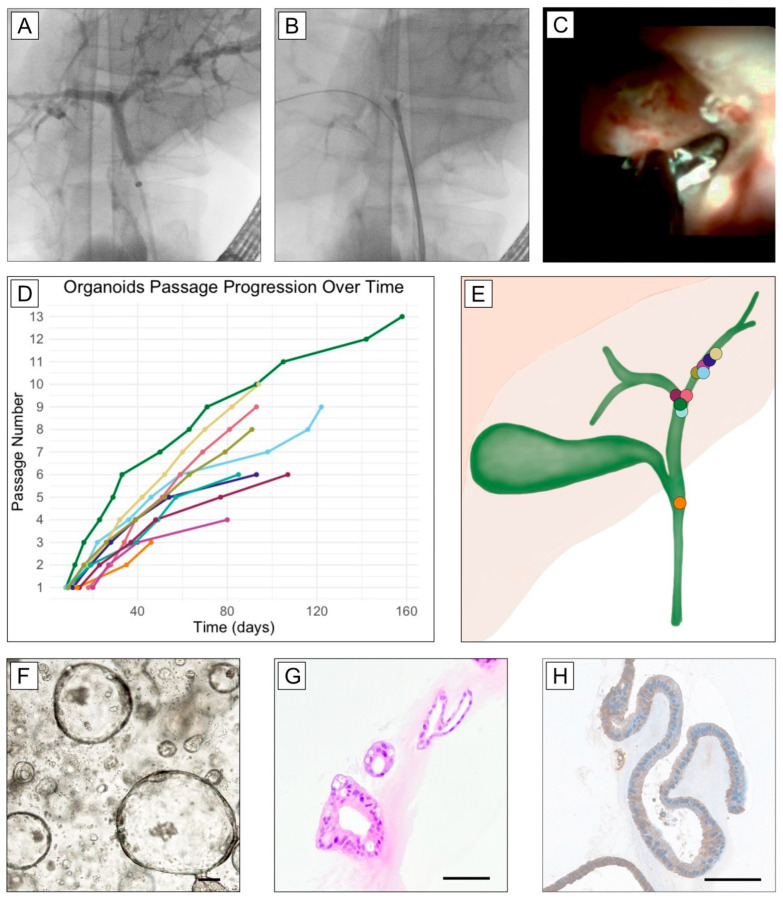
Exemplary images of an endoscopic retrograde cholangiography (ERC) of a main stricture site at the hepatic duct bifurcation after a balloon dilatation (**A**) and cholangiography-guided biopsy at the main stricture site (**B**), as well as a cholangioscopy-guided biopsy of a main stricture (**C**). Timeline of ERC-organoid passages (**D**). Graphical representation of ERC sampling sites to obtain ERC-derived organoids. The organoids show similar growth pattern regardless of the sampling site (**E**). Representative images of ERC-derived organoids: bright-field live microscopy image (**F**), hematoxylin–eosin staining (**G**), and immunohistochemistry staining (**H**) for cytokeratin-7 after formalin fixation and paraffin embedding. Scale bar = 100 μm.

First, the main stricture was identified during the cholangioscopy. After the routine biopsies for clinical use were performed, two additional biopsy specimens from a previously identified main stricture were obtained for this study. An organoid culture could be successfully accomplished in 10 out of the 10 (100%) cases. The organoids were passaged regularly for a median of six times (range: 3–13, median: 93 days, range: 36–158) before being frozen. Every organoid was stored at −196 °C and thawed at least twice. After the thawing process, all organoids remained vital for at least 15 days. Every organoid line could be passaged for the first time after 8–20 days (Figure 1D). All organoids from different patients appeared phenotypically identical and displayed positivity for CK7 (Figure 1G–H, Supplementary Table S7). In one case, a sample was replated on day one in culture because of too-dense cellularity. The left hepatic duct (LHD) was sampled most frequently (n = 5, 50%), followed by hepatic duct bifurcation (HDB, n = 4, 40%). In one case, the biopsies were obtained from the common bile duct (CBD), just below the cystic duct junction. In one case, the PSC diagnosis could not be definitively confirmed, and the patient was lost to follow-up. In two out of ten samples, we could identify deleterious mutations (Supplementary Table S8).

### 3.2. Establishment of a Patient-Derived Organoid Biobank from Liver Tumors

In parallel to the samples from PSC patients, we collected biopsy-like samples from patients undergoing surgery for liver tumors. A total of 14 organoid lines were successfully established from eight patients with primary cholangiocarcinoma, two patients with hepatocellular carcinoma (HCC), and three patients with liver metastasis from colorectal malignancies (CRCm). The patients’ characteristics and site of sampling are summarized in Table 2.

The success rate of establishing a long-term organoid line (≥10 passages) was 60% for CRCm (3/5), 57% for cholangiocarcinoma (8/14), and 33% for HCC (2/6). Every organoid line could be passaged after 7–22 days, stored at −196 °C, and thawed at least twice.

### 3.3. A Patient-Derived Organoid Model Can Be Used to Test Chemotherapy Regimens in Different Liver Malignancies

**Figure 2:**
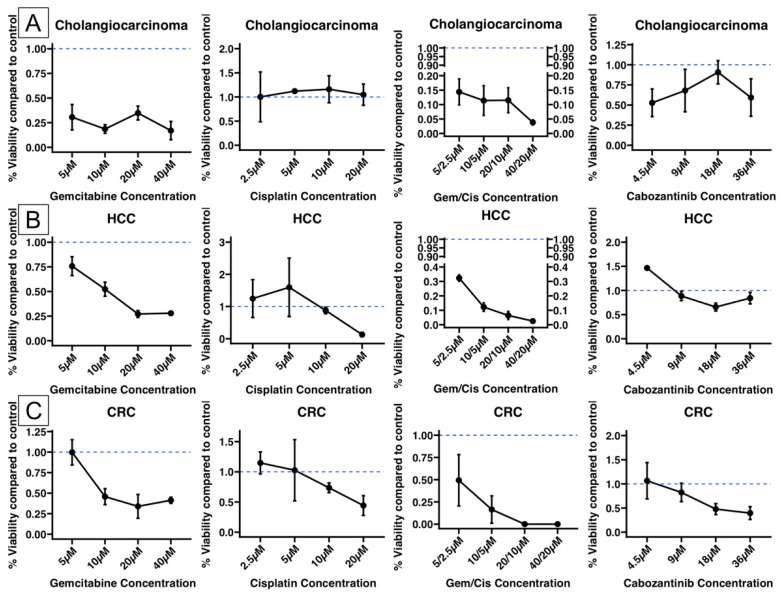
Viability after 48 h of chemotherapy testing on organoids from (**A**) cholangiocarcinoma, (**B**) hepatocellular carcinoma (HCC), and (**C**) colorectal metastasis (CRC). The mean viability and standard error of the mean are displayed. Viability is normalized to the control (Gem/Cis: gemcitabine/cisplatin).

To determine whether the organoid model responds to chemotherapy in a way that suggests its usability for real-life testing, we performed an exploratory chemotherapy test on PDOs from different liver tumors using different agents. In one CCA organoid, a decrease in viability was observed after 48 h of treatment with gemcitabine and the combination of gemcitabine and cisplatin. However, cisplatin alone did not affect viability, while cabozantinib treatment resulted in a moderate viability reduction. The patient was given oral capecitabine. After 15 months of follow-up, he had no signs of relapse (Figure 2A). In one HCC organoid, a similar decrease in viability was observed after treatment with gemcitabine alone and in combination with gemcitabine and cisplatin. Some effects were also observed after treatment with cisplatin and cabozantinib at higher concentrations (Figure 2B). The patient was enrolled in a trial and did not receive any chemotherapy. After 12 months of follow-up, no signs of relapse were observed. In the colorectal organoid, a decrease in viability was observed after treatment with gemcitabine and cisplatin, alone or in combination, and after treatment with cabozantinib. The patient did not receive any adjuvant treatment and was disease-free after 24 months of follow-up (Figure 2C).

### 3.4. Baricitinib Reduces IL-6 and MCP1 Secretion in Cholangiocarcinoma and May Have an Effect on PSC Cholangiocytes’ Secretion

**Figure 3:**
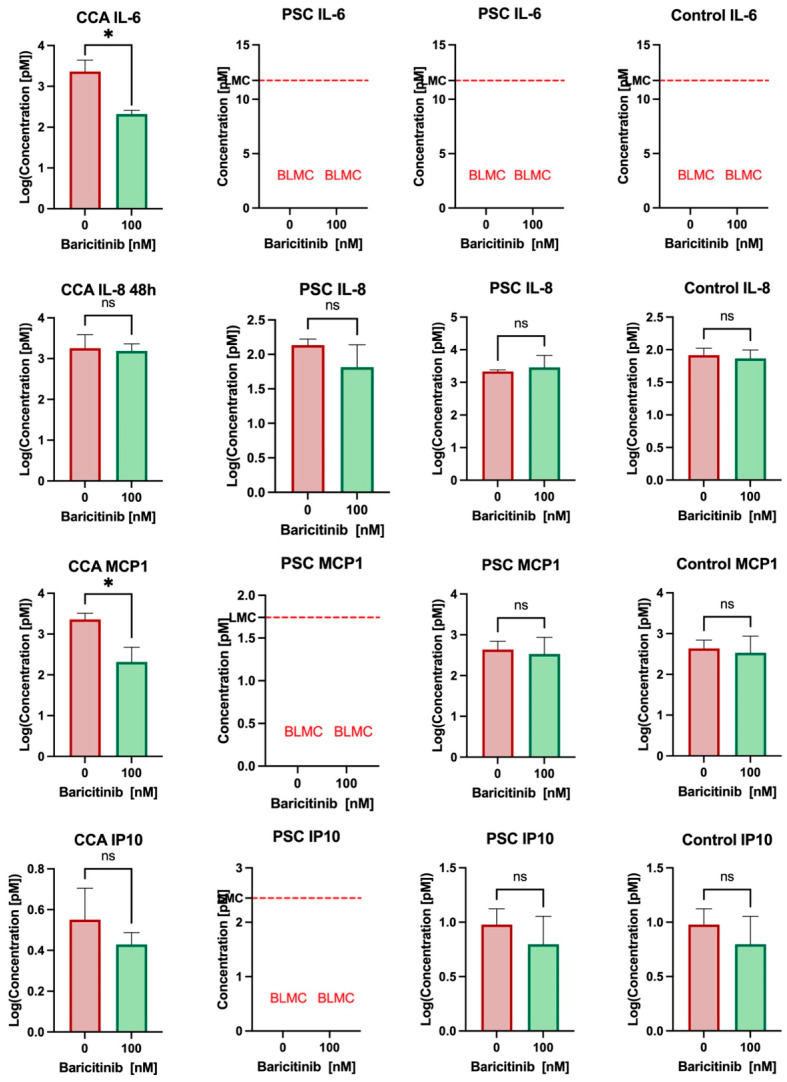
Bar plots displaying the log (concentration) of various cytokines after 48 h of treatment with baricitinib. Resulkts from one CCA organoid, two different PSC organoids and a control are shown. (BLMC: below lowest measurable concentration; CCA: cholangiocarcinoma; PSC: primary sclerosing cholangitis; ns: not significant) The means with standard deviations are displayed. (*) indicates *p* value < 0.05.

To assess the potential of the organoid model for PSC and cholangiocarcinoma in novel therapy testing, we investigated the effect of baricitinib, a JAK inhibitor, on cytokine secretion. In cholangiocarcinoma, a significant reduction in IL-6 secretion was observed in the supernatant after 48 h of incubation (log concentration 3.37 vs. 2.32, *p* = 0.038). The concentration of MCP1 was also significantly reduced after 48h of treatment (log concentration: 3.36 vs. 2.31, *p* = 0.034) (Figure 3). However, baricitinib had no effect on other cytokines, as IP-10 and IL-8 concentrations remained stable after 48 h of treatment. In the PSC organoid model, a slight decrease in IL-8 and IP-10 concentrations was detected following baricitinib treatment, though this reduction was not statistically significant. The IL-6 concentration in the supernatant of ERC-derived organoids was below the lowest measurable concentration. No significant alterations were observed in the secretion of other cytokines after treatment.

### 3.5. STAT3 Is Highly Expressed in the Tumor and Immune Microenvironment of Cholangiocarcinoma

**Figure 4:**
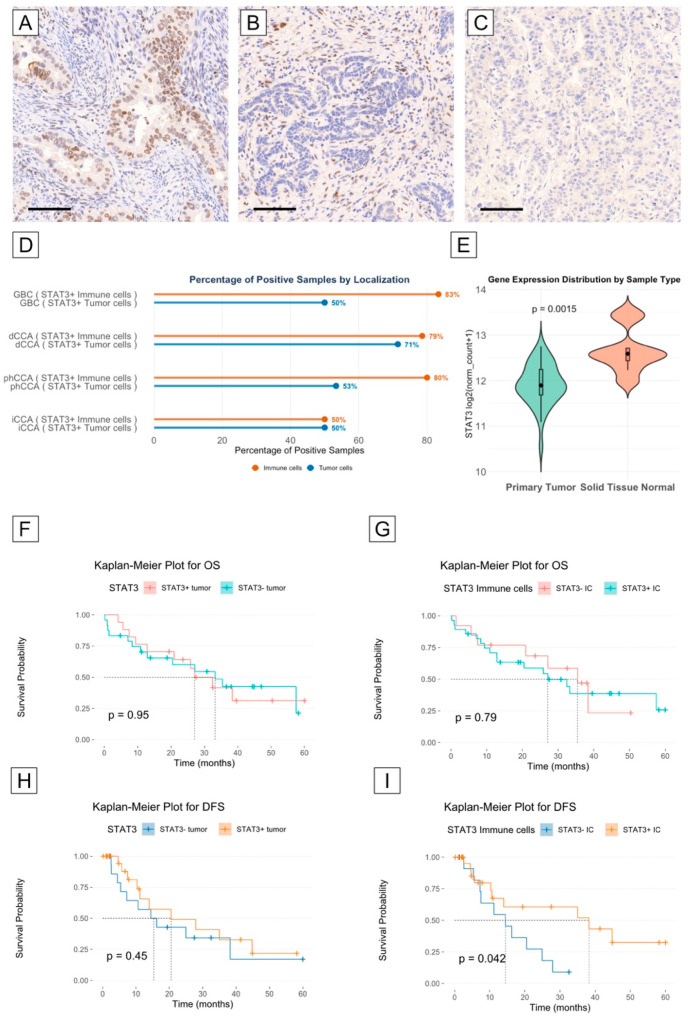
Exemplary images of STAT3+ tumor cells (**A**), STAT3+ immune cells (**B**), and STAT3– tumor cells in the immune microenvironment (**C**). Bar graphs representing the percentage of STAT3+ tumor cells (blue bars) and STAT3+ immune cells (orange bars), grouped by CCA localization (**D**). Violin plot of gene expression distribution in CCA tumor samples (green) and normal adjacent tissue (orange) from the TGCA-Chol cohort. Median, interquartile range (IQR), and smallest/largest observation greater than or equal to lower/upper hinge—1.5 × IQR (**E**). Kaplan–Meier plot for overall survival according to STAT3 immunoreactivity (low vs. high) in tumor cells (**F**) and in immune cells (**G**). Kaplan–Meier plot for disease-free survival according to STAT3 immunoreactivity (low vs. high) in tumor cells (**H**) and in immune cells (**I**). (GBC: gallbladder cancer; dCCA: distal cholangiocarcinoma; phCCA: perihilar cholangiocarcinoma; iCCA: intrahepatic cholangiocarcinoma; OS: overall survival; DFS: disease-free survival). Scale bar = 100 μm.

To determine STAT3 expression in cholangiocarcinoma, we downloaded and analyzed data from the TGCA cohort bile duct cancer CHOL (n = 45; 36 primary tumor and 9 normal tissue samples) from the platform XENA [42]. STAT3 is highly expressed in both tumor and normal tissues, and STAT3 expression was expressed at a significantly higher concentration in non-neoplastic tissue samples compared to tumor tissue samples (mean log2 expression: 12.66; IQR: 12.4–12.7 vs. 11.91, IQR: 11.68–12; *p* = 0.0015) (Figure 4E). To evaluate the STAT3 expression at the protein level, we analyzed our BTC cohort after IHC staining. In most samples, STAT3 protein expression was detected both in the tumor compartment (56%, n = 31) and in the immune microenvironment (69%, n = 38) (Supplementary Table S9). The lowest rate of positive STAT3 tumor samples was found in the iCCA group and in the GBC subgroup (50%), while the highest rate was found in the dCCA group (71%). The difference was not statistically significant (*p* = 0.7) (Figure 4D). The rate of STAT3 immune cell positivity was higher in GBC, dCCA, and phCCA compared to iCCA, but the difference was not statistically significant (83%, 80%, and 79%, respectively, vs. 50%, *p* = 0.2) (Figure 4D, Supplementary Table S9).

### 3.6. STAT3 Expression May Correlate with Longer Disease-Free Survival in Cholangiocarcinoma Patients

To explore whether STAT3 expression has a role as a prognostic factor, we first compared the overall and disease-free survival (OS and DFS) curves of patients with STAT3+ and STAT− tumor compartments, as well as with a STAT3+ and STAT− immune cell microenvironment. Patients’ characteristics according to STAT3 expression are summarized in Supplementary Tables S10 and S11. While we did not observe a difference in OS and DFS between patients with or without STAT3 expression in the tumor compartment, we did observe a significant difference in DFS between patients with STAT3+ and STAT3− expression in the immune cell microenvironment. In fact, the median DFS was longer for patients with a STAT3+ immune cell microenvironment compared to patients with a STAT3– immune cell microenvironment (38.2 vs. 14.5 months, log-rank test *p* = 0.042) (Figure 4F–I). To analyze the role of confounders, we built a Cox proportional hazards model, which ultimately did not confirm the role of STAT3+ expression in the immune microenvironment as an independent prognostic factor for DFS in our cohort (Supplementary Table S12).

## 4. Discussion

In this study, we successfully established an organoid model from biopsies obtained from PSC patients through endoscopic retrograde cholangiography. We were able to maintain long-term cultures of cholangiocyte organoids from all samples. In addition, we used this model to test a JAK inhibitor. The ability to maintain long-term cholangiocyte organoid cultures from all patient samples highlights the reproducibility of this approach. Furthermore, we demonstrated the feasibility of using this model for novel therapeutic approaches by evaluating the effects of baricitinib as a proof of concept. Organoids provide a powerful tool for disease modeling and personalized medicine because they retain key characteristics of the original tissue, including cellular heterogeneity and functionality [34,43,44]. This is particularly relevant for PSC and other biliary diseases, including CCA, where patient-derived organoids can better recapitulate in vivo responses to treatment and could serve as an intermediate step between in vitro drug screening and in vivo validation [34,45,46,47,48]. Previous studies have demonstrated the feasibility of generating reliable organoid models from bile samples, bile duct biopsies, and tissue samples from PSC patients [45,49,50,51,52]. These models of PSC that rely on bile fluid and brush cytology may not accurately reflect the localized inflammatory and fibrotic processes occurring at the site of dominant strictures. One of the key strengths of our study is the use of cholangioscopy-guided biopsies of the main strictures of PSC patients to establish organoid cultures. This allows for targeted sampling of areas most affected by chronic inflammation and fibrosis. Previous models derived from bile fluid or donor tissue may not fully represent the cellular and molecular landscape of diseased bile ducts. Our minimally invasive approach captures site-specific epithelial changes within PSC main strictures. In our study, we used endoscopic retrograde cholangiography (ERC) followed by cholangioscopy in nine out of ten cases to precisely identify the location of the main stricture. This enhances the translational relevance of the model and provides a robust platform to study PSC biology, early tumorigenesis and response to therapeutics. The rationale for targeting main strictures in PSC patients stems from their increased risk of malignancy. While strictures in PSC are often benign due to chronic inflammation and fibrosis, they are also the primary sites where cholangiocarcinoma (CCA) can develop [53,54]. By performing cholangioscopy-guided biopsies at the identified main stricture, the aim is to enhance the diagnostic relevance and ensure that the organoids reflect the pathological characteristics of high-risk biliary lesions. With this method, we identified high-risk mutations in two out of ten samples. This approach is more invasive than simple bile sampling, which does not require more biopsies than clinically necessary. However, we did not observe any biopsy-related complications, such as perforation and bleeding, in our cohort.

In our study, we measured the cytokine concentration in the supernatant from the organoids of PSC and CCA samples to determine if the method is suitable for testing novel therapies like baricitinib. Baricitinib is a first-generation, non-selective JAK inhibitor that has been approved for chronic inflammatory conditions like rheumatoid arthritis and colitis ulcerosa [55]. PSC is characterized by chronic inflammation, a condition that may also be driven by activation of the JAK/STAT3 pathway, as demonstrated by recent studies [56,57,58]. Currently, no approved medication exists for PSC that can modify disease progression or improve overall survival. Therefore, in vivo exploration of novel therapeutic targets remains pivotal.

Aberrant activation of STAT3 has been observed in several cancers, including cholangiocarcinoma [59]. Chronic inflammation is a known risk factor for the development of cholangiocarcinoma; STAT3 is involved in signaling pathways associated with inflammation, and its activation may contribute to the inflammatory microenvironment that promotes tumorigenesis [60]. In our cohort, we found expression of STAT3 protein in most tumors (53%). Interestingly, in a larger proportion of tumors (69%), the immune cells of the tumor microenvironment expressed the STAT3 protein. STAT3 positivity in the immune cell compartment was found to be associated with longer disease-free survival in our cohort.

STAT3 has been associated with pro-tumorigenic activities in many cancers. In our cohort, we found that STAT3 in the immune cell compartment was positively associated with better disease-free survival. However, in our cohort, STAT3 was not identified as an independent prognostic factor, suggesting that its impact on DFS may be influenced by additional factors. In particular, the distribution of tumor subtypes may be an important confounding factor in our study. Specifically, the STAT3+ group in our cohort contained a lower proportion of intrahepatic cholangiocarcinoma (iCCA), which generally have a poorer prognosis compared to extrahepatic subtypes. This imbalance may have contributed to the observed association between STAT3 positivity in the immune microenvironment and improved disease-free survival. Therefore, this prognostic finding should be interpreted considering this potential confounding factor. Although previous studies have associated STAT3 expression, particularly in tumor cells, with adverse clinical outcomes, our results associate STAT3 expression in the immune microenvironment with better outcomes. This may reflect a distinct biological role of STAT3 in immune regulation rather than tumor proliferation or invasion, providing a possible explanation for the observed discrepancy with the existing literature. In fact, the IL-6/STAT3 pathway has been shown to have anti-tumorigenic effects in certain conditions [61]. Therefore, it is plausible that STAT3 activation in immune cells (such as T cells, dendritic cells, and macrophages) may enhance rather than suppress anti-tumor immune responses.

Mutation analysis in liver tumor organoids revealed mutations in key cancer-associated genes, such as TP53 and KRAS, supporting their relevance for studying key molecular drivers of biliary tract cancers and drug response modeling. Interestingly, two PSC-derived organoids also harbored mutations in KRAS and TP53, which are typically associated with malignant transformation. The presence of these mutations in non-malignant PSC tissue may indicate a higher risk of progression to cholangiocarcinoma in these patients. This finding underscores the potential utility of organoid-based genomic screening to identify high-risk individuals and guide personalized surveillance strategies.

To assess whether the effect of a JAK inhibition can be tested in our CCA organoid model, we treated an organoid with baricitinib. Interestingly, we found a significant reduction in IL-6 and MCP-1 secretion after 48 h of treatment. IL-6 is a stress-response-related cytokine that has been found to be elevated in serum samples of CCA patients [62]. Previous studies have suggested that inhibition of the IL-6 signaling pathway may represent a potential therapeutic strategy in CCA [63,64]. MCP-1 (also known as CCL2, chemokine (C-C motif) ligand 2) overexpression has been linked to tumor growth, angiogenesis, and poor prognosis across multiple cancer types, and elevated serum MCP-1 correlates with tumor stages in various malignancies [65]. In addition, there is evidence that STAT3 activation can lead to MCP-1 overexpression in some tumors, creating a positive feedback loop, thereby facilitating cancer progression [66]. A recent study found that neutralization of CC2 can improve survival and remodel the tumor microenvironment in isocitrate dehydrogenase 1 (IDH1)-mutant cholangiocarcinoma (CCA), a highly aggressive sub-type of CCA [67]. Thus, JAK inhibition could represent an alternative therapeutic option for targeting the IL-6/JAK/STAT3 and the CCL2 signaling pathways.

IL-6 secretion was undetectable in PSC-derived organoids both before and after baricitinib treatment. This may reflect an inherently low basal level of IL-6 production in PSC cholangiocytes, which is consistent with their non-malignant nature, in contrast to the elevated inflammatory signaling observed in CCA. Alternatively, the lack of detectable IL-6 may be due to technical limitations, with cytokine concentrations falling below the assay detection threshold. Further studies, for example, with inflammatory stimulation or more sensitive detection methods, will be required to clarify this issue.

In addition, we performed exploratory tests with commonly used agents to assess the feasibility of using the organoid model for drug screening. Although the number of organoid lines was limited, we observed a variable but consistent decrease in viability following treatment in different liver tumor-derived organoids. These preliminary findings support the potential of patient-derived organoids as a platform for personalized therapeutic testing, although further validation in larger cohorts and in vivo models is required.

Despite these promising findings, several limitations should be considered. First, our PSC organoid model does not recapitulate immune components of the microenvironment. Future studies should explore the integration of co-cultured organoid systems with fibroblasts or immune cells to improve physiological relevance. In addition, we did not assess STAT3 expression in our patient-derived organoid models, particularly in relation to chemosensitivity and cytokine secretion. Future studies will evaluate whether STAT3-positive organoids exhibit differential responses to therapeutic agents or altered cytokine profiles, potentially providing a predictive biomarker for treatment response. Finally, while we tested a single JAK inhibitor, future investigations should be expanded to evaluate combination therapies or other targeted agents.

## Figures and Tables

**Table 1:** Patients’ characteristics of the ERC-organoid cohort.

Sample Number	Age at ERCP	Sex	Age at First Diagnosis	Sampling	Amsterdam Score	Dominant Stricture	Dysplasia	Cancer	Diagnosis	Pathology Notes
1	64	F	49	HDB	3	HDB	0	0	PSC	/
2	49	M	17	LHD	3	LHD	0	0	PSC	increased focal IG4 Plasma cells
3	49	M	48	LHD	2	LHD	0	0	PSC	/
4	34	F	34	HDB	2	HDB	0	0	PSC	/
5	47	F	33	LHD	3	LHD	0	0	PSC	/
6	27	M	21	LHD	3	LHD	0	0	PSC	/
7	42	M	42	HDB	3	HDB	0	0	PSC	/
8	41	M	23	LHD	3	LHD	0	0	PSC	/
9	49	F	47	HDB	/	HDB	0	0	Benign stricture	/
checked10	39	F	39	CBD	2	CBD	0	0	PSC	/

HDB: hepatic duct bifurcation; LHD: left hepatic duct; CBD: common bile duct; PSC: primary sclerosing cholangitis.

**Table 2:** Patient characteristics of the tumor-derived organoid cohort.

Age	Sex	Tumor Type	Sampling Site	T	N	M	G	L	V	Pn	R
61	M	phCCA	Segment III	pT2b	pN1	M0	G2	L1	V0	Pn0	R0
72	F	iCCA	Segment V	pT3	pN0	M0	G2	L0	V1	Pn1	R1
68	M	phCCA	Segment IVa	pT2b	pN0	M0	G2	L0	V0	Pn1	R1
82	F	HCC	Segment III	pT2	pNx	M0	G3	L0	V0	Pn0	R0
73	F	iCCA	Segment IVa	1b	pN0	M0	G2	L0	V0	Pn0	R1
76	M	CRC Met	Segment III	pT4a	pN0	M1	G2	L0	V0	Pn0	R0
78	M	HCC	Segment VIII	pT2	pN0	M0	G3	L0	V1	Pn0	R0
75	M	dCCA	CBD	pT3	pN0	M0	G2	L0	V0	Pn0	R0
75	M	HCC	Segment II	pT2	pNx	M0	G2	L0	V1	Pn0	R0
42	F	CRC Met	Segment V	ypT2	ypN0	M2	NA	L0	V0	Pn0	R0
80	F	phCCA	Segment III	pT1a	pN0	M0	G1	L0	V0	Pn0	R0
73	M	CRC Met	Segment V	pT2	pN0	M2	NA	L0	V0	Pn0	R0
60	M	HCC	Segment IVb	pT3	pN0	M0	G2	L0	V1	Pn0	R0

phCCA: perihilar cholangiocarcinoma; iCCA: intrahepatic cholangiocarcinoma; HCC: hepatocellular carcinoma; dCCA: distal cholangiocarcinoma; CRC Met: colorectal cancer metastasis; CBD: common bile duct.

## Data Availability

The datasets used and analyzed during the current study are available from the corresponding author on reasonable request.

## Supplementary Materials

The following supporting information can be downloaded at: https://www.mdpi.com/article/10.3390/biomedicines13051083/s1, Table S1: Wash medium composition; Table S2: Digestive medium composition; Table S3: Basic organoid medium composition; Table S4: Organoid isolation media composition; Table S5: Organoid expansion media composition; Table S6: Organoid tumoroid medium composition; Table S7: ERC-derived organoid immunoreactivity for Cytokeratin-7 after IHC staining (CK7: Cytokeratin-7); Table S8: Mutations found in the PSC organoid cohort and CCA organoids cohort; Table S9: STAT3 expression in tumor and immune cell microenvironment of the cholangiocarcinoma cohort; Table S10: BTC patients´ cohort divided in subgroup according to tumor cell STAT3 expression (positive vs negative) 1: Median (25% percentile, 75% percentile); n (%); 2: Wilcoxon rank sum test; Pearson’s Chi-squared test; Fisher’s exact test; Table S11: BTC patient cohort divided in subgroup according to tumor cell STAT3 expression (positive vs. negative) 1: Median (25% percentile, 75% percentile); n (%); 2: Wilcoxon rank sum test; Pearson’s Chi-squared test; Fisher’s exact test; Table S12: Multivariate analysis for disease-free survival in the CCA cohort.

## Abbreviations

The following abbreviations are used in this manuscript:

PSC	Primary sclerosing cholangitis
BTC	Biliary tract cancer
CCA	Cholangiocarcinoma
iCCA	Intrahepatic cholangiocarcinoma
phCCA	Perihilar cholangiocarcinoma
dCCA	Distal cholangiocarcinoma
GBC	Gallbladder cancer
ERC	Endoscopic retrograde cholangiography
EMT	Epithelial-to-mesenchymal transition
FFPE	Formalin-fixed paraffin-embedded
ECO	extrahepatic cholangiocyte organoid
JAK	Janus kinase
IL-x	Interleukin
MCP-1	Monocyte chemoattractant protein 1
TNM	Tumor, node, metastasis
TMA	Tissue microarray
H&E	Hematoxylin–eosin
ECO	Extrahepatic cholangiocyte organoid
HCC	Hepatocellular carcinoma
CRCm	Colorectal metastasis
TGCA	The Cancer Genome Atlas
IDH1	Isocitrate dehydrogenase 1
STAT3	Signal transducer and activator of transcription 3
IBD	Inflammatory bowel disease
OS	Overall survival
DFS	Disease-free survival
