# Supplementary material for: The IL-6/JAK/STAT3 Axis in Cholangiocarcinoma and Primary Sclerosing Cholangitis: Unlocking Therapeutic Strategies Through Patient-Derived Organoids
Source: Biomedicines. 2025 Apr 29;13(5):1083. doi: 10.3390/biomedicines13051083 (PMC12108797; doi:10.3390/biomedicines13051083)
Supplement: Supplementary file 1 [file biomedicines-13-01083-s001.zip › biomedicines-3555978-supplementary.pdf]

Supplemetary materials

Supplementary Table S1

Product	Manufacturer	Ref.
DMEM/F12 Medium (500 ml)	Gibco	11550446
1% GlutaMAX	Gibco	35050061
10 mM HEPES	Carl Roth	9157.1

Supplementary Table S1: Wash medium composition.

Supplementary Table S2

Product	Manufacturer	Ref.
RPMI 1640 (10 ml)	GIBCO	21875158
2% Pen/Strep	GIBCO	15140-122
250 Collagenase IV	Rockland	MB-12-100
30 U DNAse I	Thermo-Fisher	EN0521

Supplementary Table S2: Digestive medium composition

Supplementary Table S3

Product	Manufacturer	Ref.
DMEM/F12 Medium (500 ml)	Gibco	11550446
(2x) 1% Penicillin/Streptomycin	Gibco	15140122
(2x) 1% GlutaMAX	Gibco	35050061
(2x) 10 mM HEPES	Carl Roth	9157.1
(2x) 1:50 B27 supplement (without Vitamin A)	Gibco	12587010
(2x) 1:100 N2 supplement	Gibco	17502048
(2x) 10 nM recombinant human [Leu15]-Gastrin I	Merck	05-23-2301

Product	Manufacturer	Ref.
(2x) 1.25 mM n-Acetyl-L-cysteine	ThermoFisherScientific	160280500
(2x) 10 mM nicotinamide	Sigma-Aldrich	N3376

Supplementary Table S3: Basic organoid medium composition.

Supplementary Table S4. Organoid isolation media composition

Product	Manufacturer	Ref.
Basic Organoid Medium (25ml)		
L-Wnt3a conditioned media (15ml)		
R-Spondin1 conditionend media (10 ml)		
25 ng/ml recombinant Human Noggin Protein	Thermo Fisher Scientific	PHC1506
50 ng/ml recombinant human EGF	PeproTech	AF-100-15-500UG
100 ng/ml recombinant human FGF10	PeproTech	AF-100-26-500UG
25 ng/ml recombinant human HGF	PeproTech	100-39H
10 μM Forskolin	Biogems	6652995
5 μM A8301	Sigma-Aldrich	SML0788-5MG
10 μM Y-27632 Dihydrochloride	Biogems	1293823
100 μg/mL Normocin	InvivoGen	ant-nr-1

Supplementary Table S4: Organoid isolation media composition.

Supplementary Table S5. Organoid expansion media composition

Product	Manufacturer	Ref.
Basic Organoid Medium (25ml)		
R-Spondin1 conditionend media (25 ml)		
50 ng/ml recombinant human EGF	PeproTech	AF-100-15-500UG
100 ng/ml recombinant human FGF10	PeproTech	AF-100-26-500UG
25 ng/ml recombinant human HGF	PeproTech	100-39H
10 μM Forskolin	Biogems	6652995

Product	Manufacturer	Ref.
5 µM A8301	Sigma-Aldrich	SML0788-5MG
100 µg/mL Normocin	InvivoGen	ant-nr-1

Supplementary Table S5: Organoid expansion media composition.

Supplementary Table S6

Product	Manufacturer	Ref.
Basic Organoid Medium (50ml)		
50 ng/ml recombinant human EGF	PeproTech	AF-100-15-500UG
100 ng/ml recombinant human FGF10	PeproTech	AF-100-26-500UG
25 ng/ml recombinant human HGF	PeproTech	100-39H
10 µM Forskolin	Biogems	6652995
5 µM A8301	Sigma-Aldrich	SML0788-5MG
10 µM Y-27632 Dihydrochloride	Biogems	1293823
3 nM Dexamethasone		

Supplementary Table S6: Organoid tumoroid medium composition

Supplementary Table S7

Sample Number	CK7
1	strong postitivity
2	strong postitivity
3	strong postitivity
4	strong postitivity
5	strong postitivity
6	strong postitivity
7	strong postitivity
8	strong postitivity
9	strong postitivity
10	strong postitivity

Supplementary Table S7: ERC-derived organoid immunoreactivity for Cytokeratin-7 after IHC staining (CK7: Cytokeratin-7).

Supplementary Table S8

Sample	Gene	HGVS coding sequence name	HGVS protein sequence name	AF
PSC 2	KRAS	NM_033360.2:c.35G>A	NP_203524.1:p.Gly12Asp	13.9
PSC 6	P53	NM_000546.5:c.*1175A>C	(3_prime_UTR_variant)	51.4
phCCA 1	KRAS	NM_033360.2:c.34G>T	NP_203524.1:p.Gly12Cys	26
iCCA 1	KRAS	NM_033360.2:c.35G>A	NP_203524.1:p.Gly12Asp	51.7
HCC 1	TP53	NM_000546.5:c.840A>T	NP_000537.3:p.Arg280Ser	15
CRC met 1	KRAS	NM_033360.2:c.35G>T	NP_203524.1:p.Gly12Val	99.7

Sample	Gene	HGVS coding sequence name	HGVS protein sequence name	AF
CRC met 2	KRAS	NM_033360.2:c.35G>T	NP_203524.1:p.Gly12Val	63.9
CRC met 2	PIK3CA	NM_006218.2:c.1633G>A	NP_006209.2:p.Glu545Lys	63.5
CRC met 2	TP53	NM_000546.5:c.*1175A>C	(3_prime_UTR_variant)	52.9

Supplementary Table S8: Mutations found in the PSC organoid cohort and CCA organoids cohort.

Supplementary Table S9

STAT3 Expression in Tumor and Immune Cell Microenvironment						
Protein Expression	Overall N = 55	iCCA N = 20	phCCA N = 15	dCCA N = 14	GBC N = 6	p-value
STAT3+ Tumor cells	31 (56%)	10 (50%)	8 (53%)	10 (71%)	3 (50%)	0.7
STAT3+ Immune cells	38 (69%)	10 (50%)	12 (80%)	11 (79%)	5 (83%)	0.2

Supplementary **Table S9: STAT3 expression in tumor and immune cell microenvironment of the cholangiocarcinoma cohort. (phCCA: pe**rihilar cholangiocarcinoma, iCCA: intrahepatic cholangiocarcinoma; HCC: hepatocellular carcinoma, dCCA: distal cholangiocarcinoma).

Supplementary Table S10

BTC patients´ characteristics according to STAT3 expression in the tumor compartment.

Variable	STAT3- Tumor N = 24[1]	STAT3+ Tumor N = 31[1]	p-value[2]
Age	66 (58, 71)	70 (60, 76)	0.11
Sex			0.5
Male	11 (46%)	17 (55%)	
Female	13 (54%)	14 (45%)	
Tumor_Localisation			0.7
iCCA	10 (42%)	10 (32%)	
phCCA	7 (29%)	8 (26%)	
dCCA	4 (17%)	10 (32%)	
GBC	3 (13%)	3 (9.7%)	
Tumor_Grading			0.3
G1/2	18 (75%)	19 (61%)	
G3/4	6 (25%)	12 (39%)	
Tumor			>0.9
pT1-2	13 (54%)	17 (55%)	

Variable	STAT3- Tumor N = 24[1]	STAT3+ Tumor N = 31[1]	p-value[2]
pT3-4	11 (46%)	14 (45%)	
Nodes			0.7
N0	12 (50%)	17 (55%)	
N+	12 (50%)	14 (45%)	
Metastasis			>0.9
M0	21 (88%)	28 (90%)	
M+	3 (13%)	3 (9.7%)	
ASA			0.4
ASA2	6 (25%)	9 (31%)	
ASA3	17 (71%)	16 (55%)	
ASA4	1 (4.2%)	4 (14%)	
Chemotherapy			0.3
Adjuvant chemotherapy	14 (61%)	13 (45%)	
No Adjuvant chemotherapy	9 (39%)	16 (55%)	
Ca19_9	77 (38, 296)	76 (32, 701)	>0.9

Supplementary Table S10: BTC patients´ cohort divided in subgroup according to tumor cell STAT3 expression (positive vs negative) 1: Median (25% percentile, 75% percentile); n (%); 2: Wilcoxon rank sum test; Pearson's Chi-squared test; Fisher's exact test.

Supplementary Table S11

BTC patients´ characteristics according to STAT3 expression in the immune cells compartment.

Variable	STAT3- IC N = 17[1]	STAT3+ IC N = 38[1]	p-value[2]
Age	67 (63, 72)	66 (60, 73)	0.9
Sex			0.8
Male	9 (53%)	19 (50%)	
Female	8 (47%)	19 (50%)	
Tumor_Localisation			0.2
iCCA	10 (59%)	10 (26%)	

Variable	STAT3- IC N = 17[1]	STAT3+ IC N = 38[1]	p-value[2]
phCCA	3 (18%)	12 (32%)	
dCCA	3 (18%)	11 (29%)	
GBC	1 (5.9%)	5 (13%)	
Tumor_Grading			0.7
G1/2	12 (71%)	25 (66%)	
G3/4	5 (29%)	13 (34%)	
Tumor			0.3
pT1-2	11 (65%)	19 (50%)	
pT3-4	6 (35%)	19 (50%)	
Nodes			0.5
N0	10 (59%)	19 (50%)	
N+	7 (41%)	19 (50%)	
Metastasis			0.2
M0	17 (100%)	32 (84%)	
M+	0 (0%)	6 (16%)	
ASA			0.7
ASA2	6 (35%)	9 (25%)	
ASA3	10 (59%)	23 (64%)	
ASA4	1 (5.9%)	4 (11%)	
Chemotherapy			0.9
Adjuvant chemotherapy	8 (50%)	19 (53%)	
No Adjuvant chemotherapy	8 (50%)	17 (47%)	
Ca19_9	49 (32, 128)	88 (36, 1,549)	0.3

Supplementary Table S11: BTC patient cohort divided in subgroup according to tumor cell STAT3 expression (positive vs negative) 1: Median (25% percentile, 75% percentile); n (%); 2: Wilcoxon rank sum test; Pearson's Chi-squared test; Fisher's exact test.

Supplementary Table S12

Variable	Label	Estimate	Conf.low	Conf.high	P value
STAT3_IC	STAT3 IC -				
	STAT3 IC +	0.56	0.18	1.78	0.32
Tumor	pT1-2				
	pT3-4	1.38	0.45	4.25	0.58
Nodes	N0				
	N+	1.22	0.44	3.33	0.70
Metastasis	M0				
	M+	0.55	0.09	3.24	0.51
ASA	ASA2				
	ASA3	0.87	0.31	2.39	0.78
	ASA4	0.00	0.00	Inf	1.00

Supplementary Table S12: Multivariate analysis for disease-free survival in the CCA cohort.
